# Supplementary material for: Sources of path integration error in young and aging humans
Source: Nat Commun. 2020 May 26;11:2626. doi: 10.1038/s41467-020-15805-9 (PMC7250899; doi:10.1038/s41467-020-15805-9)
Supplement: Supplementary file 1 — Supplementary Information [file 41467_2020_15805_MOESM1_ESM.pdf]

SUPPLEMENTARY INFORMATION

Sources of path integration error in young and aging humans

M. Stangl*, I. Kanitscheider*, M. Riemer, I. Fiete**, T. Wolbers**

* These authors contributed equally

** These authors jointly supervised this work

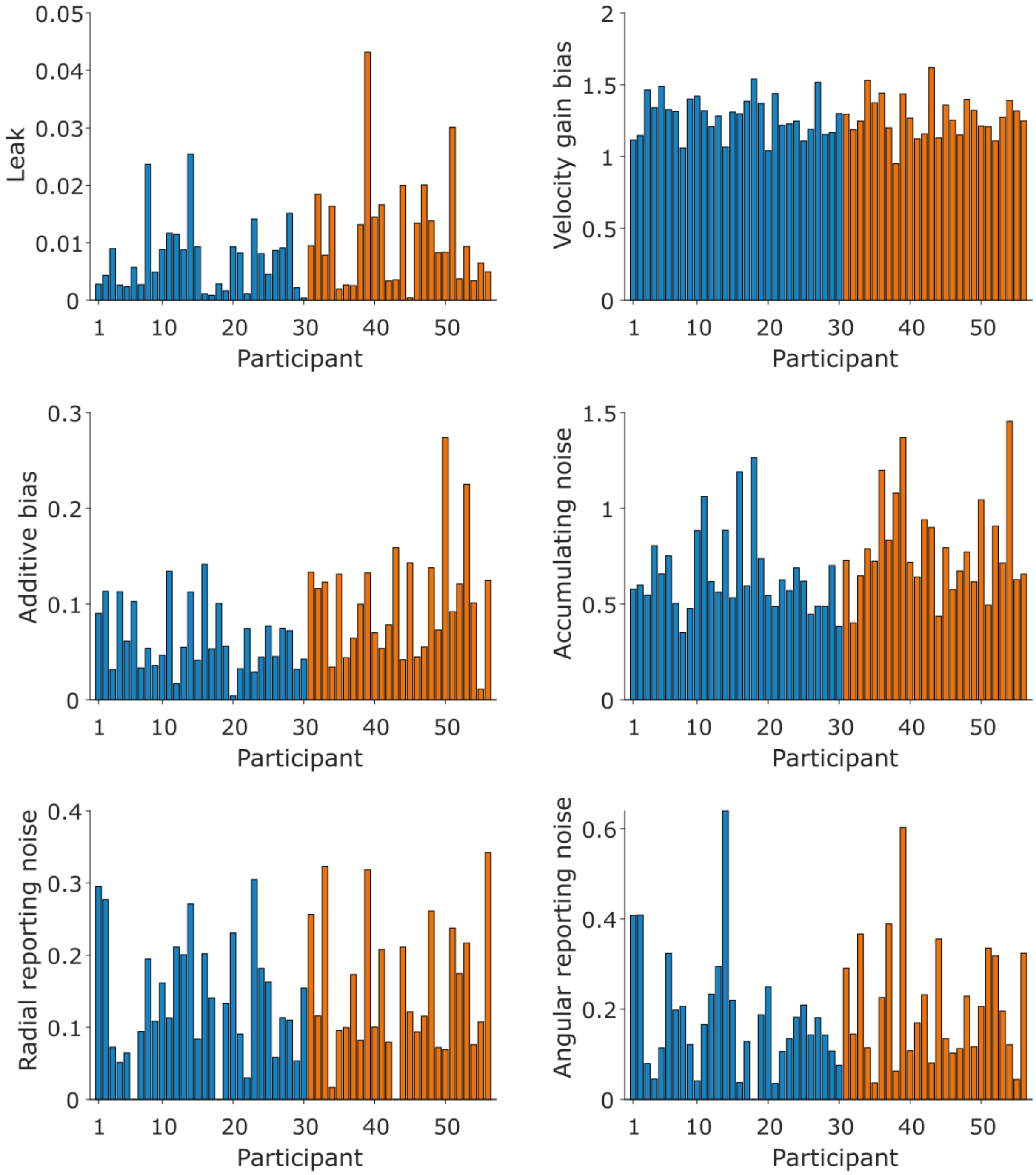

Supplementary Figure 1: Individual parameter values for single participants.

Model parameter fit of the full model, shown for each participant individually. Blue bars indicate young participants, orange bars indicate older participants. Participant ordering is identical across plots.

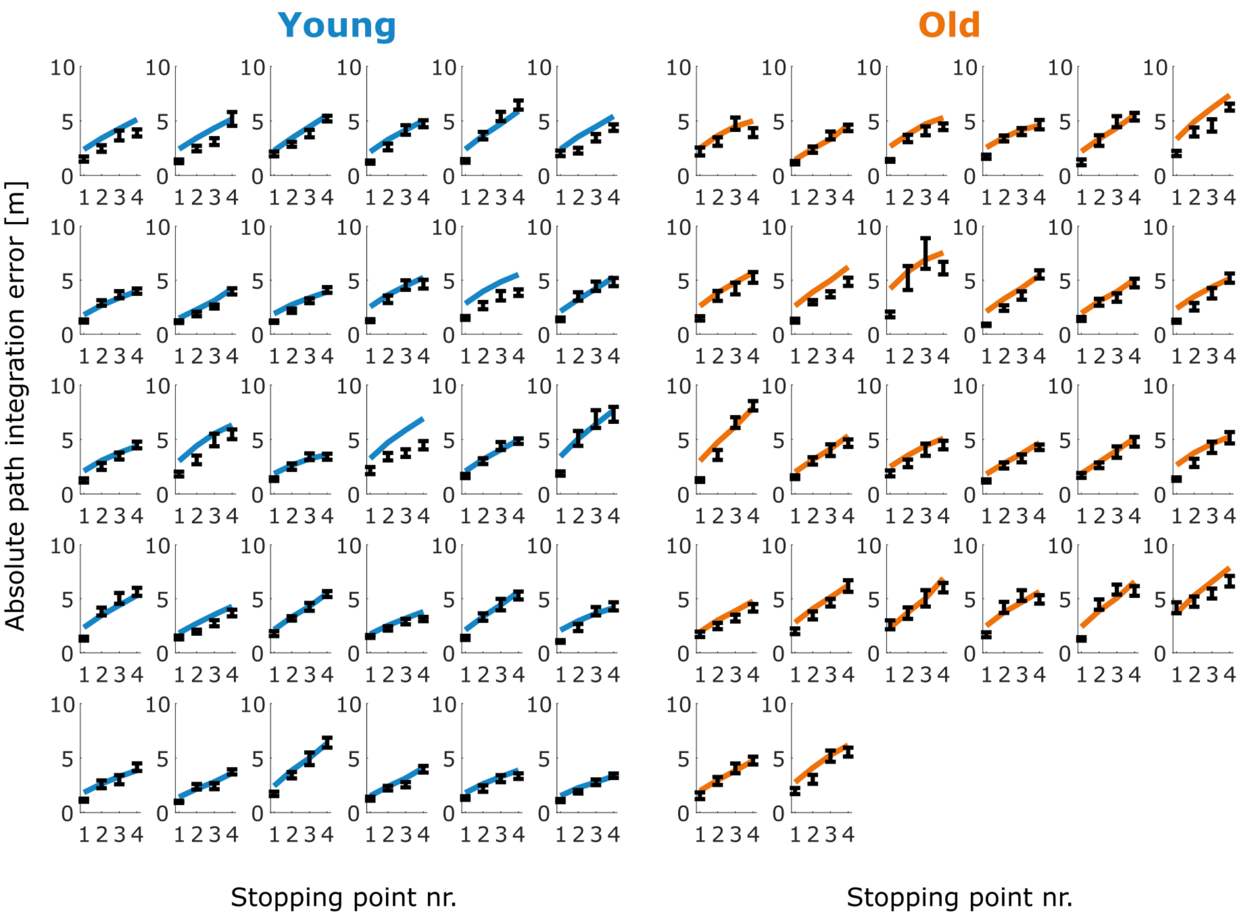

Supplementary Figure 2: Path integration errors of young and older adults versus errors predicted by the model.

Path integration errors of each individual participant (black error bars) versus errors predicted by the full model (solid lines) using each participant's individual model parameters. Error bars indicate mean +/- SEM (n = 30 trials for stopping points 1-3; n = 48 trials for stopping point 4).

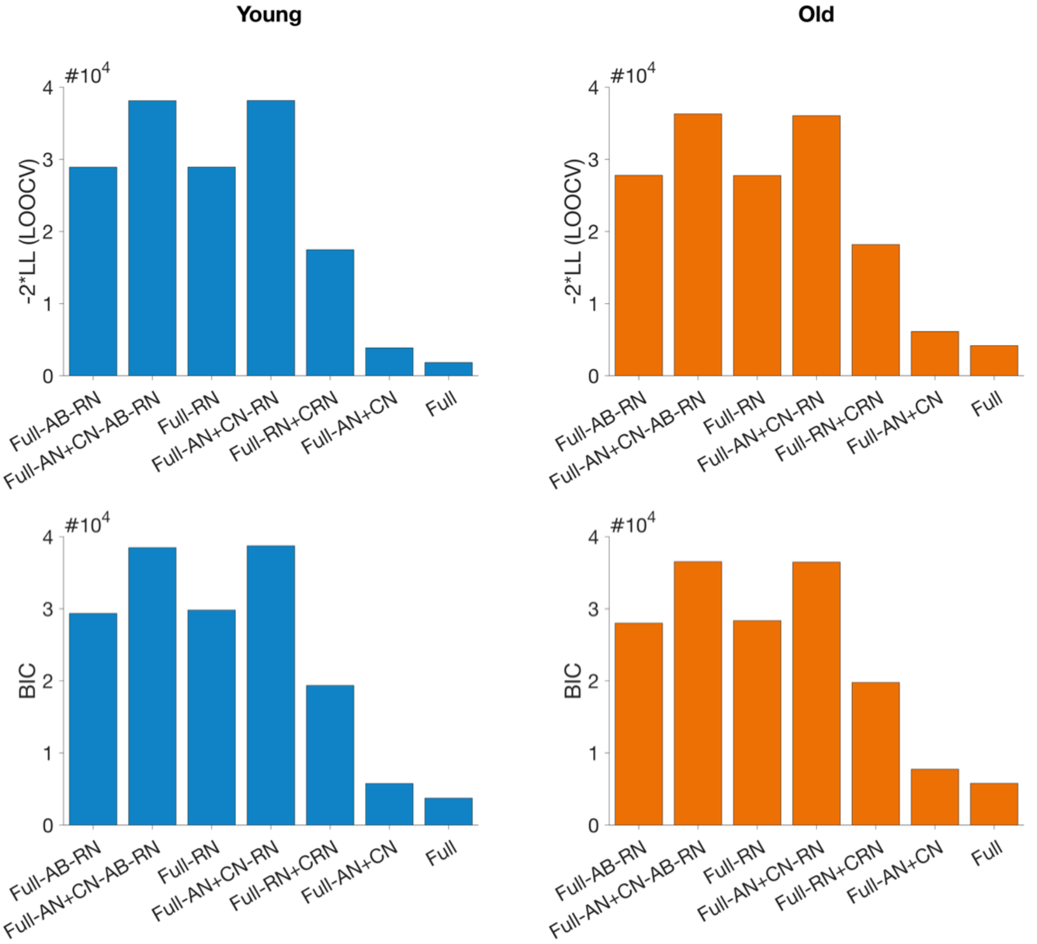

Supplementary Figure 3: Comparison between model variants using BIC and LOOCV.

Comparison between full model without additive bias, no reporting noise (Full-AB-RN); full model with constant instead of accumulating noise, no additive bias, no reporting noise (Full-AN+CN-AB-RN); full model without reporting noise (Full-RN); full model with constant instead of accumulating noise, no reporting noise (Full-AN+CN-RN); full model with constant reporting noise (Full-RN+CRN); full model with constant instead of accumulating noise (Full-AN+CN); and the default full model (Full). For both age groups, the full model was best supported by the data. Higher bars indicate poorer model-fit. More details about different model variants and BIC/LOOCV model comparisons are provided in the Methods section.

Cross-validation over trajectories:

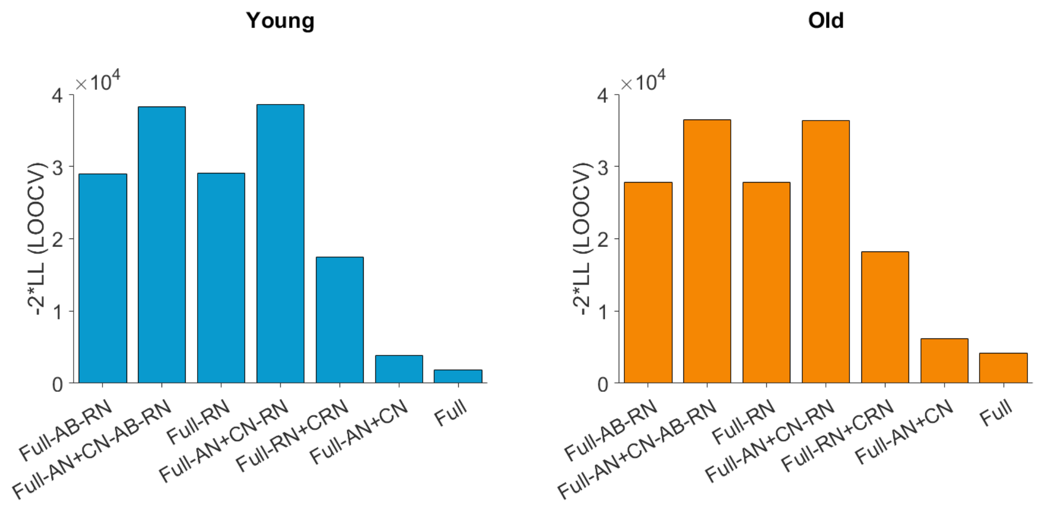

Cross-validation over stopping points:

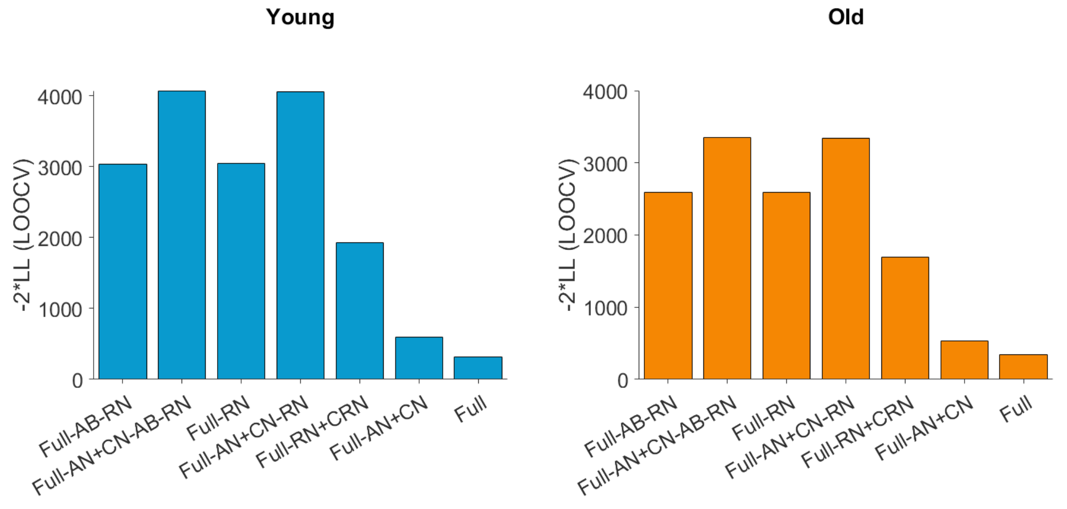

Supplementary Figure 4: Model comparison with alternative cross-validation variants.

Model comparisons using alternative cross-validation variants, in which models were trained on data from all but one trajectories and tested on the remaining trajectory (top panel), or models were trained on data from only the first three stopping points of each path and tested on the remaining fourth stopping point (bottom panel). Comparison between full model without additive bias, no reporting noise (Full-AB-RN); full model with constant instead of accumulating noise, no additive bias, no reporting noise (Full-AN+CN-AB-RN); full model without reporting noise (Full-RN); full model with constant instead of accumulating noise, no reporting noise (Full-AN+CN-RN); full model with constant reporting noise (Full-RN+CRN); full model with constant instead of accumulating noise (Full-AN+CN); and the default full model (Full). For both age groups, the full model was best supported by the data. Higher bars indicate poorer model-fit. More details about different model variants and BIC/LOOCV model comparisons are provided in the Methods section.

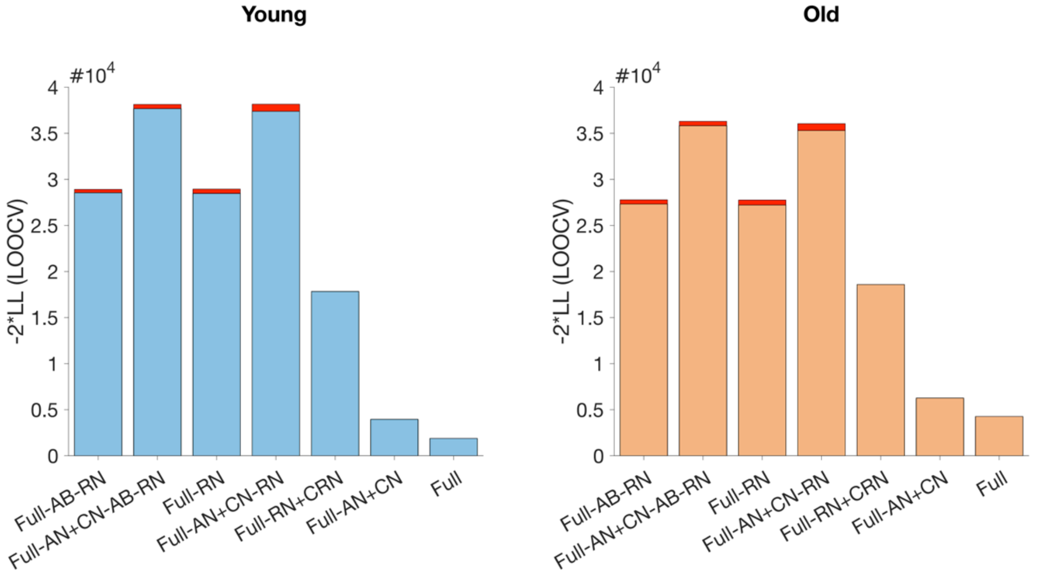

Supplementary Figure 5: Comparison of training versus test error.

Red bars indicate gap between training and test error. Comparison between full model without additive bias, no reporting noise (Full-AB-RN); full model with constant instead of accumulating noise, no additive bias, no reporting noise (Full-AN+CN-AB-RN); full model without reporting noise (Full-RN); full model with constant instead of accumulating noise, no reporting noise (Full-AN+CN-RN); full model with constant reporting noise (Full-RN+CRN); full model with constant instead of accumulating noise (Full-AN+CN); and the default full model (Full). Higher bars indicate poorer model-fit. More details about different model variants and BIC/LOOCV model comparisons are provided in the Methods section.

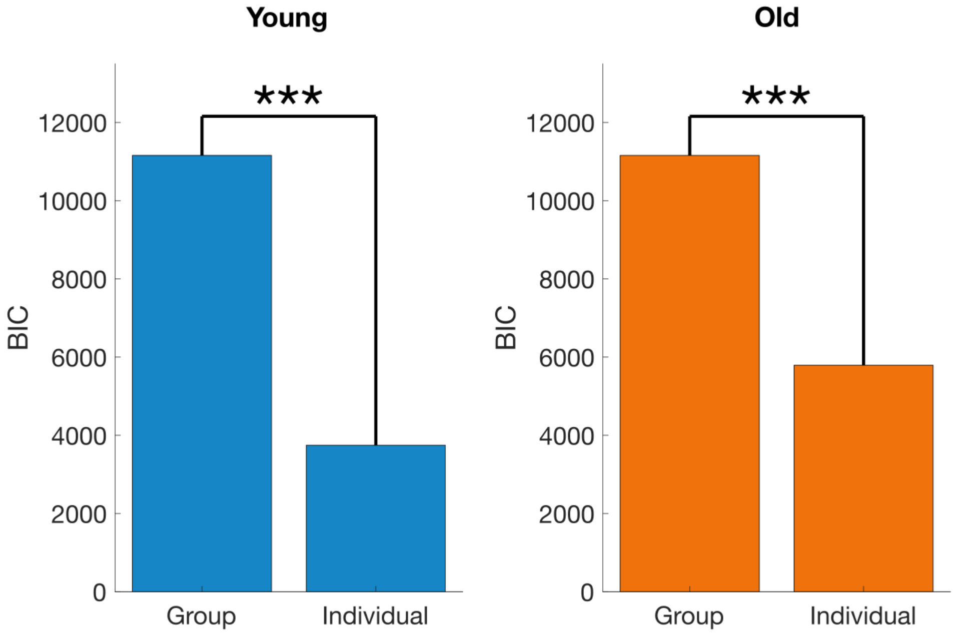

Supplementary Figure 6: Comparison between group-level and individual models.

*Model comparison using BIC between models that were fitted at the group-level and models that were fitted individually for each participant. For both age groups, the model with individual parameters per participant (i.e., the full model) was best supported by the data. Higher bars indicate poorer model-fit. *** denotes "very strong" evidence against the model with poorer model-fit ($\Delta BIC \gg 10$). More details about different model variants and BIC model comparisons are provided in the Methods section.*

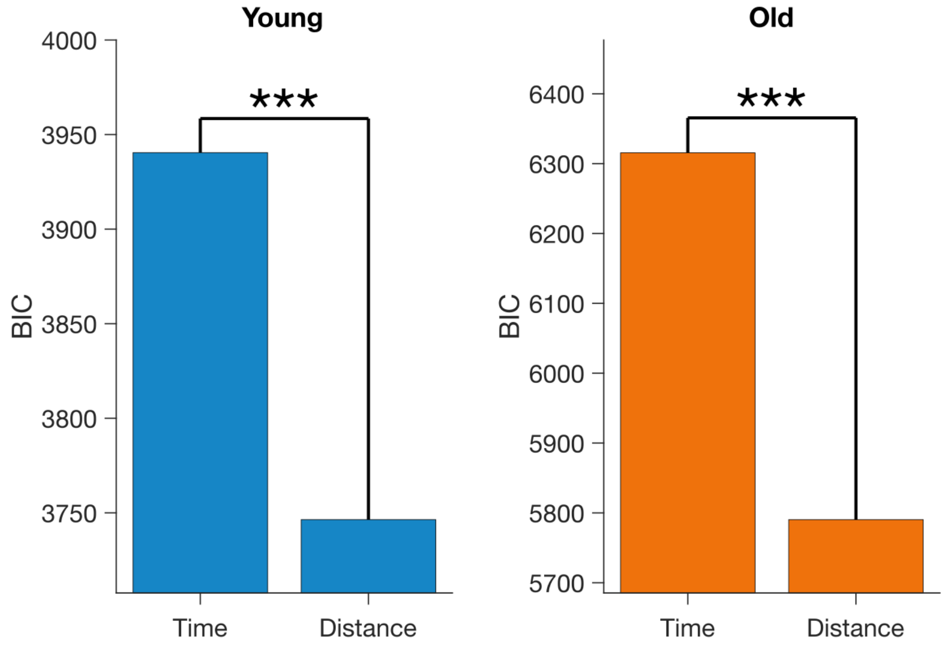

Supplementary Figure 7: Comparison between "time model" and "distance model" variant.

*Model comparison using BIC between a model with time-scaling of the internal path integration error and the full model with distance-scaling. For both age groups, the full model is better supported by the data. Higher bars indicate poorer model-fit. *** denotes "very strong" evidence against the model with poorer model-fit ($\Delta BIC \gg 10$). More details about different model variants and BIC model comparisons are provided in the Methods section.*

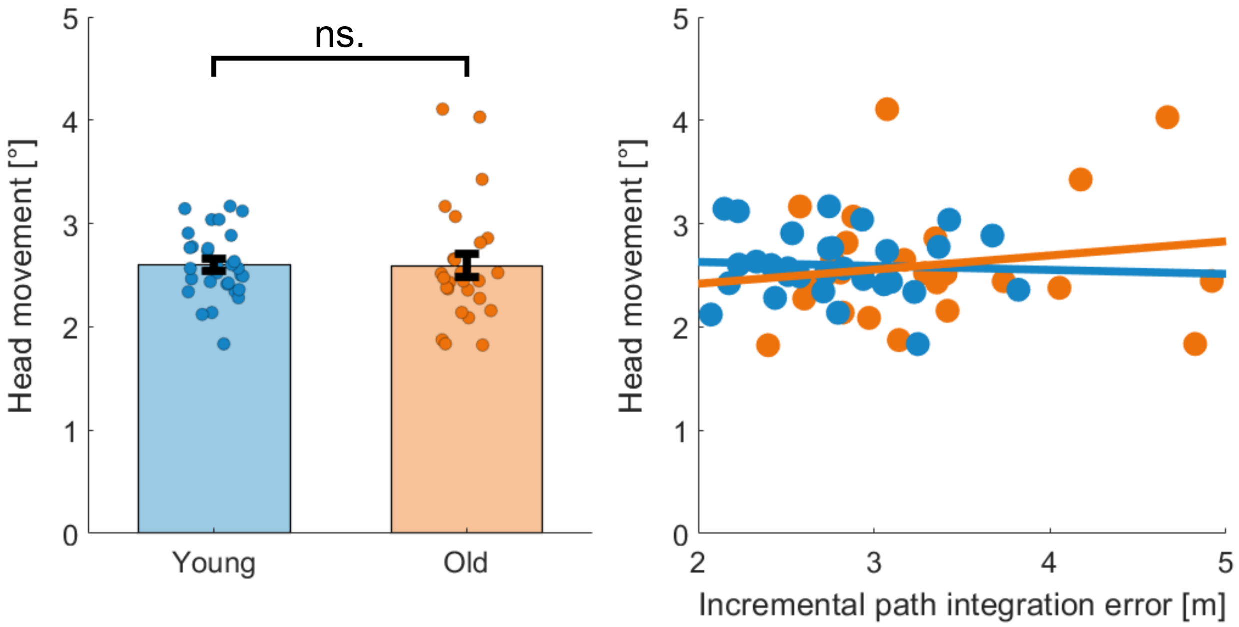

Supplementary Figure 8: Head movements during walking.

Head orientation was measured at each timepoint in 3 dimensions (yaw, pitch, roll) by the built-in gyrometer of the head mounted display. Individually for each participant, we calculated the sum of head rotations per timepoint in all three dimensions, before calculating the average degree of head movement per timepoint across all paths. The degree of head movement during walking was not significantly different between young and older adults (left panel). The degree of head movement was not correlated with individual path integration performance across both young and older adults, or within individual age groups (right panel). Error bars indicate mean +/- SEM (n = 30 young vs. 26 older participants). "ns." denotes a non-significant difference in a one-sided permutation test with 10000 permutations.

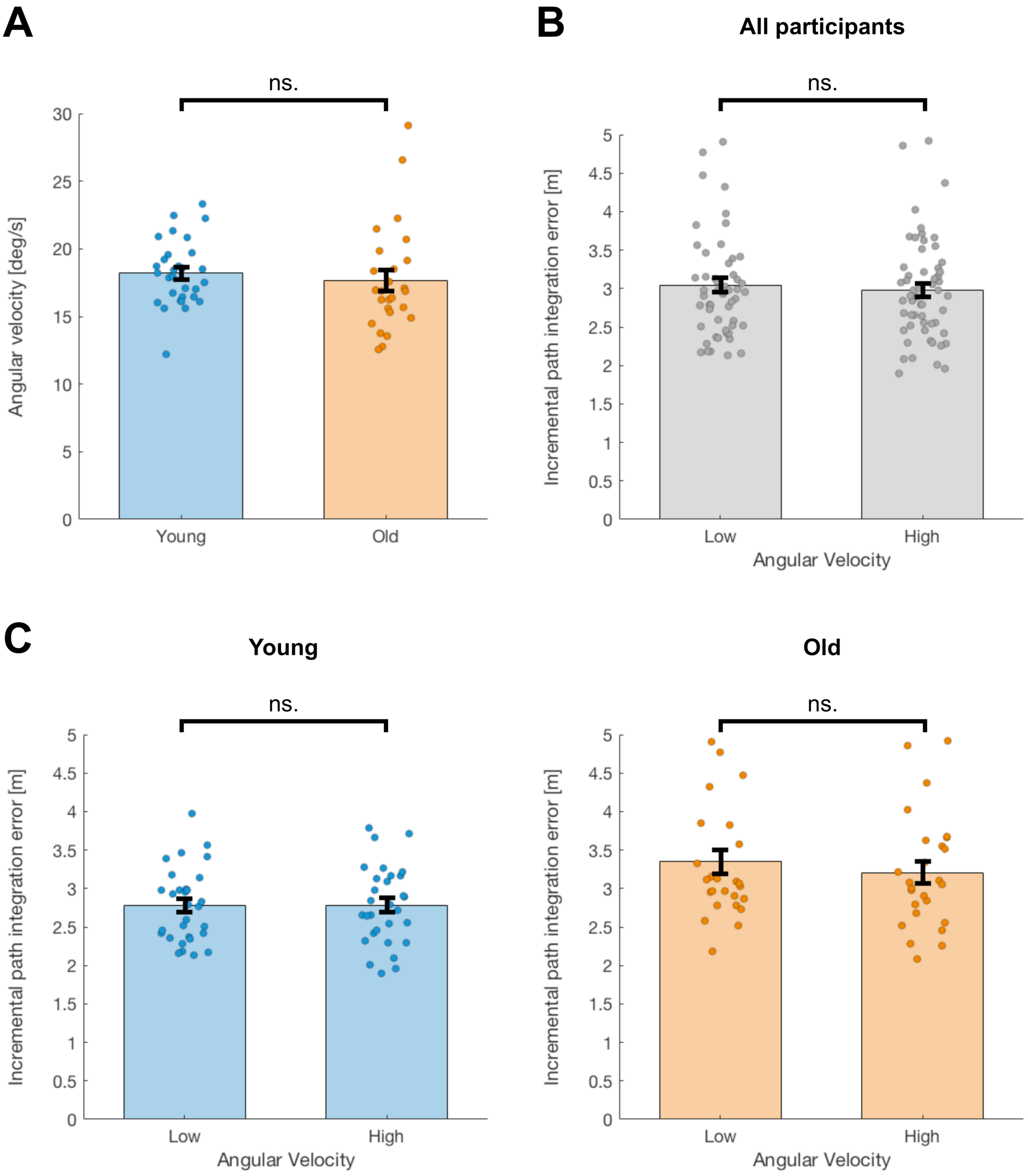

Supplementary Figure 9: Angular velocity during walking.

Angular velocity was quantified as angular displacement in the horizontal plane (i.e., yaw dimension) per timepoint. We first calculated each participant's angular velocity for each timepoint along a trajectory, before we calculated the average angular velocity for each individual path segment (i.e., separately for each walked path, from the starting point of a path to stopping point 1; between stopping point 1 and 2; and so on), as well as each participant's average angular velocity across all path segments.
A: *The participants' average angular velocity along trajectories (across all path segments) was not significantly different between young and older adults (n = 30 young vs. 26 older participants).*
B: *Across all participants, path integration error was not significantly different between path segments with lower versus path segments with higher angular velocity (n = 56 participants).*
C: *Also within the groups of young or older adults, path integration error was not significantly different between path segments with lower versus path segments with higher angular velocity (blue bars: n = 30 young participants; orange bars: n = 26 older participants).*

Error bars indicate mean +/- SEM. "ns." denotes non-significant differences. Statistical comparisons were carried out using one-sided permutation tests with 10000 permutations.

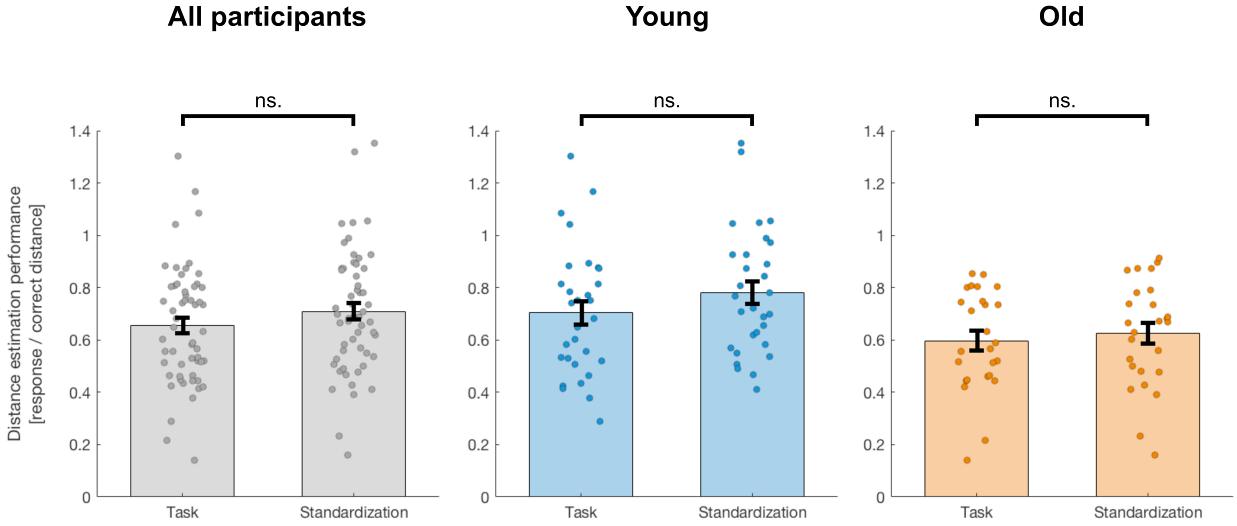

Supplementary Figure 10: Distance estimation performance for task paths versus standardization paths.

Comparison between raw (uncorrected) distance estimates during the task and distance estimates on standardization paths. To account for differences in distance lengths between task paths and standardization paths, the participants' distance estimation performance was quantified for each response separately as the ratio between the responded distance and the correct distance for a given trial. Consequently, values of 1 indicate a perfect response (response distance = correct distance), values < 1 indicate undershooting (response distance < correct distance), and values > 1 indicate overshooting (response distance > correct distance). Note that for task paths as well as for standardization paths, participants had to estimate the length of a straight distance vector, as they were asked to indicate the direct distance back to the path's starting point (rather than the total distance walked), and therefore distance estimation performance can be directly compared. Distance estimation performance was not significantly different between task and standardization paths, neither across all participants (gray bars; $p = 0.11$; $n = 56$ participants), nor within groups of young (blue bars; $p = 0.12$; $n = 30$ young participants) or older adults (orange bars; $p = 0.28$; $n = 26$ older participants). Error bars indicate mean +/- SEM. "ns." denotes non-significant differences. Statistical comparisons were carried out using one-sided permutation tests with 10000 permutations.

Supplementary Note 1

While the nature of our path integration task (i.e., participants being guided by an experimenter along pre-defined trajectories in a virtual environment) allowed for tight control of both walking behavior and sensory cues that could be used for path integration, it should be noted that this approach may have limited the ecological validity of our experiment. On the one hand, as aging is known to affect walking behavior at multiple levels (e.g. speed, veering, intermittent stopping), a systematic comparison between age groups necessitated control of walking behavior. However, the precise effects of active versus passive walking on path integration remain unclear. Previous work has shown that self-guided walking does not necessarily improve performance[1], and that actively selecting a path does not lead to behavioral benefits compared to passively following a predetermined path[2]. While these results suggest that allowing participants to walk on their own would not have significantly changed our results, the precise impact of self-guided walking on path integration mechanisms and performance remain to be determined in future studies. On the other hand, while comparable to many other studies on human path integration[3,4,5,6], the length of our trajectories is relatively short compared to trajectories typically experienced during real-world navigation. However, the signatures of the underlying neural computations should also be revealed in trajectories of smaller length. Sensing and integration of self-motion cues, updating of position and orientation estimates, and computing a homing vector are computations needed for any trajectory, independent of its length. Moreover, given that path integration inevitably accumulates error, path integration computations are particularly useful for shorter trajectories. When covering longer distances without having access to static environmental cues, humans typically accumulate large amounts of error that can even lead to complete disorientation[7]. As a consequence, positional estimates need to be frequently corrected by static environmental cues[8]. Together, these findings therefore suggest that path integration over short trajectories - as investigated in our study - is relevant for everyday navigation.

Supplementary References

1. Yamamoto, N. The role of active locomotion in space perception. *Cogn. Process.* **13**, 365–368 (2012).

2. Wan, X., Wang, R. F. & Crowell, J. A. The effect of active selection in human path integration. *J. Vis.* **10**, 25–25 (2010).

3. Klatzky, R. L. et al. Acquisition of route and survey knowledge in the absence of vision. *J. Mot. Behav.* **22**, 19–43 (1990).

4. Loomis, J. M. et al. Nonvisual navigation by blind and sighted: Assessment of path integration ability. *J. Exp. Psychol. Gen.* **122**, 73–91 (1993).

5. Péruch, P., Borel, L., Magnan, J. & Lacour, M. Direction and distance deficits in path integration after unilateral vestibular loss depend on task complexity. *Cogn. Brain Res.* **25**, 862–872 (2005).

6. Stangl, M. et al. Compromised Grid-Cell-like Representations in Old Age as a Key Mechanism to Explain Age-Related Navigational Deficits. *Curr. Biol.* **28**, 1108–1115 (2018).

7. Souman, J. L., Frissen, I., Sreenivasa, M. N. & Ernst, M. O. Walking Straight into Circles. *Curr. Biol.* **19**, 1538–1542 (2009).

8. Etienne, A. S. & Jeffery, K. J. Path integration in mammals. *Hippocampus* **14**, 180–192 (2004).
