# Supplementary material for: Sources of path integration error in young and aging humans
Source: Nat Commun. 2020 May 26;11:2626. doi: 10.1038/s41467-020-15805-9 (PMC7250899; doi:10.1038/s41467-020-15805-9)
Supplement: Supplementary file 2 — Reporting Summary [file 41467_2020_15805_MOESM2_ESM.pdf]

natureresearch

Corresponding author(s): Matthias Stangl & Ingmar Kanitscheider

Last updated by author(s): March 03, 2020

Reporting Summary

Nature Research wishes to improve the reproducibility of the work that we publish. This form provides structure for consistency and transparency in reporting. For further information on Nature Research policies, see Authors & Referees and the Editorial Policy Checklist.

Statistics

For all statistical analyses, confirm that the following items are present in the figure legend, table legend, main text, or Methods section.

n/a	Confirmed	
☐	☒	The exact sample size (*n*) for each experimental group/condition, given as a discrete number and unit of measurement
☐	☒	A statement on whether measurements were taken from distinct samples or whether the same sample was measured repeatedly
☐	☒	The statistical test(s) used AND whether they are one- or two-sided<br>*Only common tests should be described solely by name; describe more complex techniques in the Methods section.*
☐	☒	A description of all covariates tested
☐	☒	A description of any assumptions or corrections, such as tests of normality and adjustment for multiple comparisons
☐	☒	A full description of the statistical parameters including central tendency (e.g. means) or other basic estimates (e.g. regression coefficient) AND variation (e.g. standard deviation) or associated estimates of uncertainty (e.g. confidence intervals)
☐	☒	For null hypothesis testing, the test statistic (e.g. *F*, *t*, *r*) with confidence intervals, effect sizes, degrees of freedom and *P* value noted<br>*Give P values as exact values whenever suitable.*
☐	☒	For Bayesian analysis, information on the choice of priors and Markov chain Monte Carlo settings
☒	☐	For hierarchical and complex designs, identification of the appropriate level for tests and full reporting of outcomes
☐	☒	Estimates of effect sizes (e.g. Cohen's *d*, Pearson's *r*), indicating how they were calculated

Our web collection on statistics for biologists contains articles on many of the points above.

Software and code

Policy information about availability of computer code

Data collection	WorldViz Vizard 5.1 Virtual Reality Software (WorldViz LLC, www.worldviz.com)
Data analysis	MATLAB 2016b (The MathWorks, Natick, MA, USA); Curve Fitting Toolbox for MATLAB 2016b; Statistics and Machine Learning Toolbox for MATLAB 2016b; Optimization Toolbox for MATLAB 2016b; Custom MATLAB sourcecode is available from the authors upon reasonable request.

For manuscripts utilizing custom algorithms or software that are central to the research but not yet described in published literature, software must be made available to editors/reviewers. We strongly encourage code deposition in a community repository (e.g. GitHub). See the Nature Research guidelines for submitting code & software for further information.

Data

Policy information about availability of data

All manuscripts must include a data availability statement. This statement should provide the following information, where applicable:

- Accession codes, unique identifiers, or web links for publicly available datasets
- A list of figures that have associated raw data
- A description of any restrictions on data availability

The data that support the findings of this study are available for download (see Data availability section in the manuscript).

Field-specific reporting

Please select the one below that is the best fit for your research. If you are not sure, read the appropriate sections before making your selection.

☐ Life sciences ☒ Behavioural & social sciences ☐ Ecological, evolutionary & environmental sciences

For a reference copy of the document with all sections, see nature.com/documents/nr-reporting-summary-flat.pdf

Behavioural & social sciences study design

All studies must disclose on these points even when the disclosure is negative.

Study description	This is a behavioral study with human research participants and quantitative data (performance-related and from motion tracking systems). A computational model was developed and applied for data analysis.
Research sample	62 healthy humans took part in this study. They were recruited via phone from a database of the German Center for Neurodegenerative Diseases (DZNE). They had no reported history of neurological or psychiatric disease and no reported motor deficits during normal walking or standing. All participants reported right-handedness and had normal or corrected-to-normal eyesight. Prior to the study, all participants underwent the Montreal Cognitive Assessment (MoCA) screening tool for mild cognitive impairment (Nasreddine et al., 2005, Journal of the American Geriatrics Society). Participants who did not exceed a MoCA cut-off score of 23 (following Luis et al., 2009, International Journal of Geriatric Psychiatry) were excluded from the study and did not participate in any further measurements. Consequently, the data of the remaining 56 participants was used for data analyses: The group of young adults consisted of 30 participants (15 woman, 15 men) aged between 19 and 26 years (M = 22.0, SD = 2.0 years), whereas the group of older adults consisted of 26 participants (13 woman, 13 men) aged between 62 and 78 years (M = 69.0, SD = 4.6 years).
Sampling strategy	The data of 30 young adults (mean age = 22 years) and 26 older adults (mean age = 69 years) were used for data analyses. These sample sizes were chosen to be comparable to previous studies that investigated human path integration performance, age-related differences in path integration abilities, and computational models of path integration errors in humans (e.g., Allen et al., 2004, Perception & Psychophysics; Lappe et al., 2007, Experimental Brain Research; Lappe et al., 2011, Experimental Brain Research; Adamo et al., 2012, Frontiers in Aging Neuroscience; Harris & Wolbers, 2012, Hippocampus; Lakshminarasimhan et al., 2018, Neuron), in order to enable comparisons of our data with previously reported results.
Data collection	During the path integration task, participants had to repeatedly indicate the distance and orientation from an intermediate stopping point along a path to the path's starting point. The exact pose (locations and viewing orientation) of a participant was tracked in steps of 100 ms (10 Hz) throughout the task using the Vicon Motion Tracking System with 12 cameras of type T10 (Vicon, Oxford, UK). Moreover, participants saw a virtual environment via a Head Mounted Display, which enabled rotational tracking by a gyroscope, an accelerometer, and a magnetometer, and provided information about orientation per timepoint in 3 dimensions (yaw, pitch, roll), with a rotational update rate of 1000 Hz. The participants' distance estimates were reported verbally in meters and centimeters, and were manually recorded by the experimenter (typed into a spreadsheet). In addition, participants filled out pen-and-paper versions of questionnaires (such as the screening tool for mild cognitive impairment, etc.) before and after the experiment. Only one participant and one or several experimenter(s) were present in the room during the experiment. Researchers were not blind to experimental conditions and hypothesis, as well as to group allocation, as we've tested two experimental groups, young adults (mean age 22 years) and older adults (mean age 69 years), and the age difference between members of the two groups was obvious in most cases. Data analysis, however, was performed with automated MATLAB scripts that did not distinguish between data of different experimental groups.
Timing	Experimental data was collected between September and November 2015.
Data exclusions	Prior to the study, all participants underwent the Montreal Cognitive Assessment (MoCA) screening tool for mild cognitive impairment (Nasreddine et al., 2005, Journal of the American Geriatrics Society). In order to avoid that our results could be confounded by cognitive impairments of individual participants, six older adults who did not exceed a MoCA cut-off score of 23 (following Luis et al., 2009, International Journal of Geriatric Psychiatry) were excluded from the study and did not participate in any further measurements.
Non-participation	One participant dropped out, because they were not able to fully understand how the experimental task had to be performed (despite several attempts to clarify the instructions). No other participants dropped out after they had initially agreed to participate in the study.
Randomization	Participants were allocated into two experimental groups: the group of young adults (< 26 years) and the group of older adults (> 62 years). Therefore, random allocation of participants into experimental groups was not possible, but pre-determined by the participants' age. The impact of co-variates was controlled in several ways: (i) By only testing participants who had no reported history of neurological or psychiatric disease and no reported motor deficits during normal walking or standing, who reported right-handedness and had normal or corrected-to-normal eyesight. (ii) By excluding participants who did not exceed a cut-off score of 23 the Montreal Cognitive Assessment (MoCA) screening tool for mild cognitive impairment. (iii) Analytically, by demonstrating that co-variates that might potentially impact our results (such as head movements during walking and angular walking velocity) were on average not significantly different between age groups, and/or not correlated with performance of individual participants within and across age groups. (iv) Participants' response at so-called "standardization-paths" (i.e., straight lines) was used to correct for each participant's bias in converting their internal location estimate to a verbal response (see Methods for more details).

Reporting for specific materials, systems and methods

We require information from authors about some types of materials, experimental systems and methods used in many studies. Here, indicate whether each material, system or method listed is relevant to your study. If you are not sure if a list item applies to your research, read the appropriate section before selecting a response.

Materials & experimental systems

n/a	Involved in the study
☒	☐ Antibodies
☒	☐ Eukaryotic cell lines
☒	☐ Palaeontology
☒	☐ Animals and other organisms
☐	☒ Human research participants
☒	☐ Clinical data

Methods

n/a	Involved in the study
☒	☐ ChIP-seq
☒	☐ Flow cytometry
☒	☐ MRI-based neuroimaging

Human research participants

Policy information about studies involving human research participants

Population characteristics	See above.
Recruitment	Participants were recruited via phone from a database of the German Center for Neurodegenerative Diseases (DZNE).
Ethics oversight	The experiment received approval from the Ethics Committee of the University of Magdeburg.

Note that full information on the approval of the study protocol must also be provided in the manuscript.
